# Unveiling distinct kinematic profiles among total knee arthroplasty candidates through clustering technique

**DOI:** 10.1186/s13018-024-04990-8

**Published:** 2024-08-14

**Authors:** Lina Abou-Abbas, Nicola Hagemeister, Youssef Ouakrim, Alix Cagnin, Philippe Laundry, Glen Richardson, Michael J. Dunbar, Neila Mezghani

**Affiliations:** 1 Applied and Artificial Intelligence Institute (I2A), TELUQ University, Montreal, Quebec Canada; 2 Electrical ad Computer Engineering Department, Lebanese American University, Byblos, Lebanon; 3 Open Innovation Laboratory (OIL-ETS), Ecole de Technologie Superieure, Montreal, Quebec Canada; 4 Department of Systems Engineering, Ecole de Technologie Superieure, Montreal, Quebec Canada; 5 Emovi, Montreal, Quebec Canada; 6 Division of Orthopaedic Surgery, Dalhousie University, Halifax, Nova Scotia Canada

**Keywords:** Osteoarthritis, KneeKG, Clustering, K-means, Principal Component Analysis

## Abstract

****Background**:**

Characterizing the condition of patients suffering from knee osteoarthritis is complex due to multiple associations between clinical, functional, and structural parameters. While significant variability exists within this population, especially in candidates for total knee arthroplasty, there is increasing interest in knee kinematics among orthopedic surgeons aiming for more personalized approaches to achieve better outcomes and satisfaction. The primary objective of this study was to identify distinct kinematic phenotypes in total knee arthroplasty candidates and to compare different methods for the identification of these phenotypes.

****Methods**:**

Three-dimensional kinematic data obtained from a Knee Kinesiography exam during treadmill walking in the clinic were used. Various aspects of the clustering process were evaluated and compared to achieve optimal clustering, including data preparation, transformation, and representation methods.

****Results**:**

A K-Means clustering algorithm, performed using Euclidean distance, combined with principal component analysis applied on data transformed by standardization, was the optimal approach. Two unique kinematic phenotypes were identified among 80 total knee arthroplasty candidates. The two distinct phenotypes divided patients who significantly differed both in terms of knee kinematic representation and clinical outcomes, including a notable variation in 63.3% of frontal plane features and 81.8% of transverse plane features across 77.33% of the gait cycle, as well as differences in the Pain Catastrophizing Scale, highlighting the impact of these kinematic variations on patient pain and function.

****Conclusion**:**

Results from this study provide valuable insights for clinicians to develop personalized treatment approaches based on patients’ phenotype affiliation, ultimately helping to improve total knee arthroplasty outcomes.

## Introduction

Knee osteoarthritis (KOA) is a leading cause of disability among older adults, and is characterized by changes in joint structure, joint pain, mechanical joint dysfunction, and muscle weakness. It is a degenerative condition that can significantly affect the way people move, especially during weight-bearing activities such as walking [1]. Assessing the condition of patients suffering from this multifactorial disease is complex because of multiple associations between clinical, functional, and structural characteristics [2]. Knee kinematics have been largely studied in this population as KOA patients exhibit distinct kinematic patterns during gait compared to healthy individuals [3–8]. Furthermore, kinematic characteristics are associated with disease progression and patient clinical outcomes throughout the knee OA continuum of care [9, 10]. This is notably the case in total knee arthroplasty (TKA) candidates, as knee kinematics and patient outcomes presurgery may contribute to outcomes post-TKA. The interest in knee kinematics is increasing in the context of TKA where orthopedic surgeons aim towards more personalized approaches to achieve better outcomes, as up to 20% of patients remain unsatisfied post-TKA [11]. While multiple surgical techniques have emerged these past years, opposing mechanical alignment and approaches aiming at restoring native kinematic alignment, there has been little consensus on the optimal strategy (i.e., technique, implant, etc.) to adopt based on patient characteristics [12, 13]. This can partially be explained by the fact that although TKA candidates often present similarities in terms of disease severity and functional impairments, significant variability exists within this population.

The identification of phenotypes in KOA patients has gained interest in recent years as care continues to move toward personalization. This approach is based on the well-recognized heterogeneity of KOA patients and the use of clustering algorithms to partition a dataset into multiple clusters (i.e., or phenotypes) such that the similarity within each phenotype is greater than the similarity between phenotypes. In recent years, clustering techniques have been applied in various fields, including medicine, biology, and social sciences. The application of phenotype research in TKA candidates may provide valuable insights for orthopedic surgeons and help them base their choice of intervention on the patient’s phenotype affiliation. Furthermore, Spil et al. recently defined a framework dedicated to KOA phenotype research to standardize reports on such studies [14, 15]. The purpose of this study was to identify distinct phenotypes in TKA candidates based on knee kinematics following the framework of Spil et al. Furthermore, this study aimed to investigate the associations between the identified phenotypes and patient clinical characteristics.

## Materials and methods

**Fig. 1:**
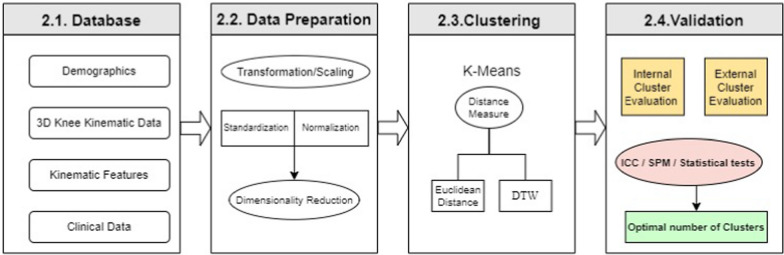
Methodology for comprehensive kinematic data analysis: A stepwise approach involving Data Preparation, Clustering using K-Means with varying distance measures (Euclidean Distance or Dynamic Time Warping), and Validation using Inter-Cluster Classification (ICC), Statistical Parametric Mapping (SPM), in addition to traditional statistical tests

The block diagram presented in Fig. 1 illustrates the different steps in the methodology used in this study to identify distinct clusters (i.e., phenotypes) from the kinematic data. This sequential methodology is designed to ensure a comprehensive and systematic approach. The first step is data collection, including the capture of kinematic, demographic and clinical data. These data are then modified through a preparation process including scaling and dimensionality reduction to reduce their complexity, making it easier to analyze and interpret them. The next step is the determination of clustering techniques aiming to identify kinematic clusters using K-means. This unsupervised machine-learning algorithm based on centroids has been largely used for analyzing kinematic trajectories through clustering [16]. The clustering process involves varying distance measures and clustering parameters, such as the number of clusters. Finally, the resulting clusters are validated for accuracy. This validation process involves using intercluster correlation and statistical hypothesis testing. The clusters, or phenotypes at this point, are evaluated based on their clinical features, demographic data and clinical information, and the results are compared to determine the accuracy of the clustering process.

### Database

The database includes demographic details (See Table 1), three-dimensional (3D) knee kinematic data, kinematic features, and clinical data from 80 candidates for total knee arthroplasty (TKA) with knee osteoarthritis (KOA) confirmed by X-ray imaging and by an experienced orthopaedic surgeon after a physical exam. This dataset was taken from a previous study (ethics approval obtained by the Nova Scotia Health Authority Research Board, reference number: NSHA ROMEO 1016253), which consisted of 178 patients with moderate to severe KOA patient referred by their general practitioner for a surgical consult. Only patients that were deemed surgical candidate for a total knee arthroplasty by the orthopaedic surgeons were kept for the current analysis. Inclusion criteria were as follows: patients with primary KOA confirmed through X-ray imaging and by an experienced orthopedic surgeon after a physical examination. Exclusion criteria included patients unable to walk on a treadmill due to neurological or balance disorders due to neurological or imbalance disorder. The cohort of TKA candidates consisted of 51 women and 29 men, providing a diverse representation of both sexes within the database.

**Table 1:** Demographic data

Variable	Mean (M)	Standard deviation (SD)
Age (years)	64.79	9.34
BMI ($\text {Kg/m}^{2}$)	33.13	7.06
Weight (Kg)	92.87	23.70
Height (m)	1.67	0.10
Pain Catastrophizing Score	17.53	13.55
Sex (Males/Females)	29/51	-

Kinematic data were captured using a Knee Kinesiography exam with the $\hbox {KneeKG}^{\text {TM}}$ system (Emovi Inc., Canada) while the patient walked on a commercial treadmill. This advanced technology is designed to provide accurate 3D measurements of dynamic knee alignment, offering objective data for individuals with movement impairments related to orthopedic conditions. The $\hbox {KneeKG}^{\text {TM}}$ system has received regulatory approvals, including FDA 510(k) clearance, Health Canada licensing, and CE marking, underscoring its reliability and compliance with medical device standards. Unlike traditional static imaging methods such as X-rays or MRI, the Knee Kinesiography exam provides insights into joint function during active movement. This dynamic assessment capability makes the $\hbox {KneeKG}^{\text {TM}}$ system a valuable tool for evaluating and managing orthopedic issues with quantifiable data [17]. The 3D kinematic data combined the knee movement in the sagittal, frontal, and transverse planes to form single-vector data of 300 raw points for each participant, with each plane containing 100 points (i.e., kinematic curves). In addition to these measures, 69 kinematic features extracted from 3D kinematic curves were also included in the database. These features were identified based on an exhaustive review of the literature and variables commonly assessed in clinical biomechanical studies in KOA populations: 30 features from the frontal plane (i.e., adduction/abduction or varus/valgus), 17 features from the sagittal plane (i.e., flexion/extension), and 22 features from the transverse plane (i.e., internal/external rotation). The features extracted included maximums, minima, angles at specific instants of gait, and ranges of motion (ROMs) throughout different phases of the gait cycle (e.g., loading, stance, swing, etc.). These features, which have been extensively studied in clinical biomechanics research are known to provide important insights into the kinematic behavior of KOA patients [2, 7].

Clinical features comprise subjective and objective data collected through patient self-administered questionnaires and supervised functional tests. The Oxford Knee Score (OKS) was used to assess pain and function in activities of daily living (ADL) through a 12-item questionnaire [18]. Based on its original publication, each question is scored from 1 to 5, so an overall OKS can be calculated, ranging from 12 (best outcome) to 60 (worst outcome). To better understand how patients experience their pain, the Pain Catastrophyizing Scale (PCS) was used to assess the tendency to magnify, ruminate, and feel helpless about pain [19]. These three aspects can be evaluated as subscores (3 features), and an overall PCS score can be calculated (the higher the score is, the more catastrophizing the patient’s pain experience is). Finally, patients were invited to perform the Timed-Up-And-Go test (TUG-test) to objectively assess their function. The TUG test starts with the patient sitting in a chair, who then raises, walks 3 ms at a comfortable pace, turns, and walks back to the chair to sit back down. The score is the time (in seconds) it takes to perform this sequence (the greater the time is, the worse is the function). Taken together, these 6 clinical features can provide a complete picture of different aspects of important outcomes in KOA patients, namely, pain (impact on ADL, magnification, and catastrophizing) and function (impact on ADL and subjective assessment).

### Data preparation

#### Data transformation methods

Amplitudes in kinematic data largely vary across the three different planes of movement (e.g., from 0 to $60-70^{\circ }$ in the sagittal plane vs. from −5 to +10$^{\circ }$ on average for the two other planes), resulting in heterogeneous data existing at different scales. To address this issue, data transformation was used to rescale the data and remove any biases due to differences in the measurement scales. By doing so, data become more comparable and easier to analyze, thus facilitating a more accurate interpretation of the knee movement patterns. Two distinct data transformation methods were tested: standardization and normalization. The normalization (also known as min-max scaler) method involves scaling the data to a fixed range of values, usually between 0 and 1, by subtracting the minimum value and dividing it by the range. On the other hand, standardization (also known as standard scaler or z-normalization) centers the data around its mean and scales it by its standard deviation, resulting in an unit variance. While normalization is advantageous for preserving the original range of the data, standardization is better suited for data with a Gaussian distribution and can handle outliers more effectively. These two methods were compared to identify the most appropriate technique for scaling the data used in this study.

#### Kinematic representation methods

Two distinct methods for representing kinematic data were then evaluated: a global representation method and a local representation method. The global approach involves utilizing a vector consisting of 300 kinematic data points. The local approach involved using the set of 69 kinematic features extracted from the 3D kinematic curves. Once again, the two approaches were compared to identify the most appropriate to identify clusters with maximal differences for this database.

#### Principal component analysis

Once this step was achieved, a dimensionality reduction phase was integrated, aiming to reduce the complexity of the process data by extracting essential features representing the variability of the data. We applied the principal component analysis (PCA) technique and evaluated its ability to capture the most important features discriminating the different kinematic clusters. PCA is a popular technique for transforming the original data into a new set of orthogonal variables called principal components [20]. These components capture the most significant sources of variability in the data and are ranked according to their contribution to the overall variance. PCA is widely used in pattern recognition and data mining because it simplifies complex datasets while retaining the essential information [21, 22].

### Clustering

#### K-means

As already described, clustering is used to identify distinct clusters by segregating data (i.e., 3D kinematic data here) into consistent groups, with the intention of extracting the knee kinematic phenotypes by averaging the kinematic curves within each group. This enables the formation of more homogeneous groups with mean patterns that are representative of each phenotype. The clustering algorithm used in this study is K-means, which is commonly used in unsupervised learning to partition data into K clusters, minimizing the sum of the squared distances between the data points and their assigned cluster centroid [23]. The process begins by selecting K centroids at random, then iteratively assigning each data point to its closest centroid and adjusting the centroid position accordingly. The algorithm terminates when the centroids no longer change, or the maximum number of iterations is reached.

#### Distance between data points

Two different methods were tested to set the optimal distance between the data points and each cluster to which they were assigned: the Euclidian distance and dynamic time warping (DTW). The Euclidean distance is a measure of the straight-line distance between two data points in an Euclidean space. It is calculated as the square root of the sum of the squared differences between the corresponding features or variables of two points. This distance metric assumes that the dimensions are independent and equally weighted, which makes it a useful option for clustering when the data are continuous and uniformly scaled. However, it may not be the best choice for datasets with high dimensionality or when the variables are not independent or equally weighted. DTW is a distance metric that is often used in time series analysis, including kinematic or motion capture data. DTW measures the similarity between two sequences of data points, even when they have different lengths and warping (i.e., when one sequence is distorted or shifted with respect to the other). It finds the optimal alignment of sequences by stretching or compressing them in time to minimize the distance between the corresponding points [24]. DTW can be useful for clustering time-series data with irregular shapes and is often used in applications such as speech recognition, gesture recognition, and motion analysis [25].

### Validation

To assess the quality of the clusters produced by the clustering algorithms, we used both internal and external cluster evaluation methods. For the internal evaluation, we calculated the intraclass correlation coefficient (ICC) to measure the similarity among the observations within each cluster. The ICC is a commonly used measure to evaluate the reliability or consistency of measurements within a single cluster and can provide insight into the internal validity of the clusters produced by the algorithm. Additionally, we employed Statistical Parametric Mapping (SPM) by [26]) to evaluate the statistical significance of the differences between the clusters in terms of the features or variables used for clustering. This approach involves analyzing 1-D continuous data without preconceived hypotheses, allowing the detection of any potential differences or patterns that may not have been previously considered by [26]. For the external evaluation, we used statistical significance tests such as Student’s t tests and ANOVAs to identify any significant differences between clusters in terms of demographic, clinical, and kinematic data. A p-value <0.05 was considered to indicate statistical significance. These two evaluation methods were also applied to determine the best number of clusters.

## Results

### Data transformation methods

**Fig. 2:**
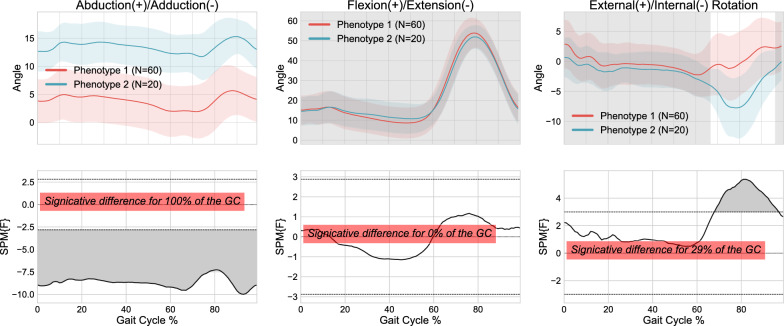
Identification of kinematic phenotypes through K-means clustering and standardization-based data rescaling, followed by 1D-SPM statistical analysis

**Fig. 3:**
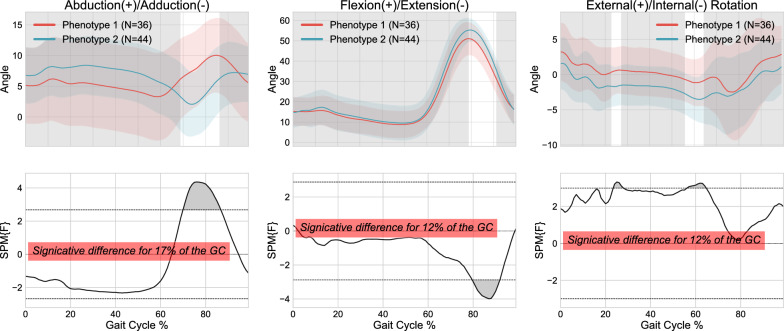
Identification of kinematic phenotypes through K-means clustering and normalization-based data rescaling, followed by 1D-SPM statistical analysis

K-means clustering algorithm was subsequently first used on the database with applied standardization (also referred to as standard scaling) and subsequently with normalization using min-max scaling to compare both transformation methods. Figures 2 and 3 illustrate the kinematic phenotypes, which were determined by averaging the 3D kinematic curves within two distinct clusters with the standardization and normalization methods, respectively. Within each figure, the second line of the graphs shows the results of the 1D-SPM statistical analysis, displaying the kinematic curve differences between clusters in each plane. The analysis revealed a significant difference throughout the entire gait cycle in the abduction/adduction plane when the standardization method was used, while this difference was observed only for 17% of the gait cycle in the same plane with the normalization method. While the results on both other planes were similar and more limited in terms of significance (i.e., difference for less than 30% of the gait cycle at maximum), the results in the abduction/adduction plane were used to choose the standardization method as the best one to scale the data.

### Kinematic representation methods

**Fig. 4:**
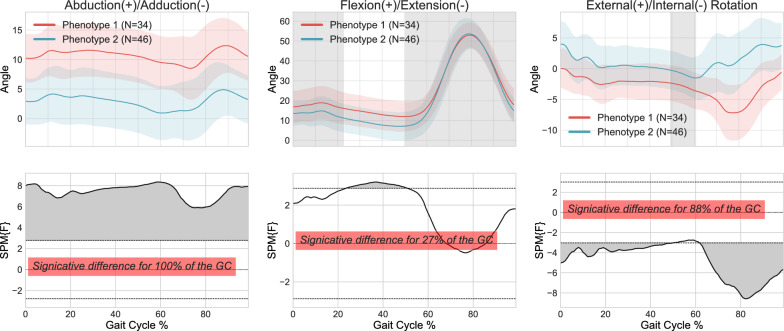
Kinematic phenotypes identified using K-means and standardization, with 1D-SPM statistical analysis using 69 features from 3D kinematic data (local representation)

**Fig. 5:**
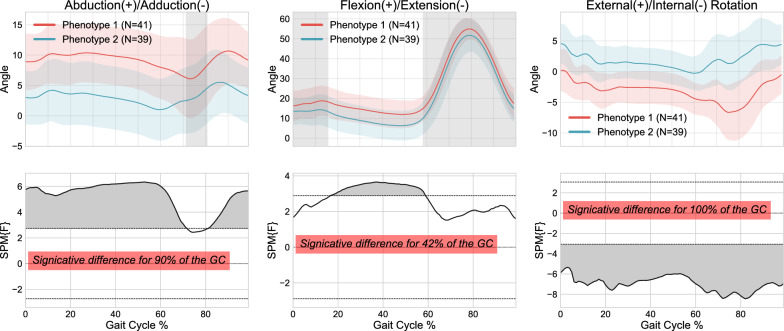
Kinematic phenotypes identified using K-means and standardization, with 1D-SPM statistical analysis using 300 data points (global representation) from 3D kinematic data using Euclidean distance

SPM differences and ICCs were calculated for both the set of 69 kinematic features (i.e., local representation) and the 300 kinematic raw points (i.e., global representation) from the sagittal, frontal, and transverse planes. According to the local representation Fig. 4, SPM analysis revealed a significant difference in abduction-adduction throughout the entire cycle, for 27% of the gait cycles in the flexion/extension plane and for 88% in the external-internal rotation plane between the two distinct clusters. On average, SPM showed significant differences between clusters using the local approach for 71.67% of the gait cycle when combining the three planes. For the global representation, an average significant difference between the two clusters was observed for 77.33% of the gait cycle (Fig. 5). The ICCs for both the local and global representations are presented in Table 2.

**Table 2:** Intraclass correlation coefficients for the local and global representations of 3D knee kinematics

ICCs	ICC Local		ICC Global	
	Cluster 1	Cluster 2	Cluster 1	Cluster 2
Abduction/adduction	0.845	0.755	0.873	0.562
Flexion/extension	0.643	0.649	0.799	0.683
External/internal rotation	0.559	0.726	0.5	0.641

Our findings revealed a statistically significant correlation within the kinematic clusters with both approaches, with ICC values ranging from 0.643 to 0.845, indicating a moderate to strong relationship. This finding implies that patients within the same cluster exhibit patterns of knee joint movement during the walking task, similar to those of patients in other clusters. Therefore, the kinematic data used in this study are consistent and reliable, further supporting the relevance of identifying kinematic phenotypes in TKA candidates. Considering the similar ICCs between both approaches, SPM differences were used to choose the global representation as a slightly more effective method to discriminate clusters and for subsequent analyses.

### Distance between data points

**Fig. 6:**
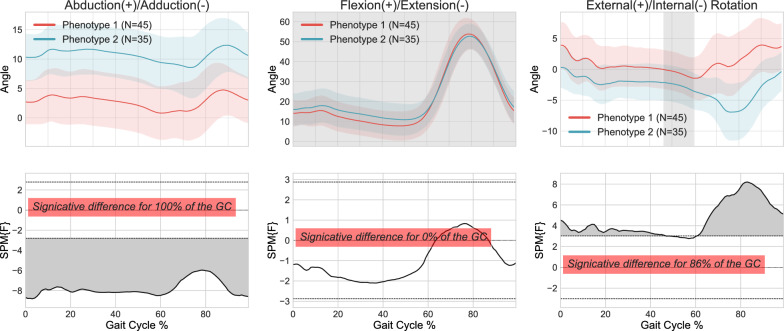
Kinematic phenotypes identified using K-means and standardization, with 1D-SPM statistical analysis using 300 data points (global representation) from 3D kinematic data using Dynamic Time Warping

Comparisons in terms of the Euclidean distance and dynamic time-warping approaches (DTW) are presented in Figs. 5 and 6 respectively.

Figure 5 shows that the Euclidean distance was significantly different throughout 90% of the gait cycle in the abduction/adduction plane, 100% of the gait cycle in the rotation plane and 42% of the gait cycle in the flexion/extension plane. The DTW results in Fig. 6 show a significant difference throughout the entire gait cycle in the abduction/adduction plane, for 86% of the gait cycles during rotation, but no significant differences were observed in flexion/extension. The ICCs for both techniques ranged from 0.5 to 0.873, demonstrating once again a significant correlation between the various knee kinematic curves within the clusters. Based on these results, the Euclidean distance method was deemed the most effective for this step of the methodology and was used for subsequent steps of the clustering process.

### Optimal number of clusters

To further improve the analysis, signal decomposition with PCA was applied on the data. Through a series of experiments,different numbers of components from 3 to 10 were tested, reaching an optimal outcome using 8 components. With this set-up for the PCA algorithm and using the most effective transformation, representation, and data-points distance methods, ICCs and SPM differences were assessed to determine the optimal number of clusters to achieve the best clustering process.Two and three clusters were subsequently identified.

**Fig. 7:**
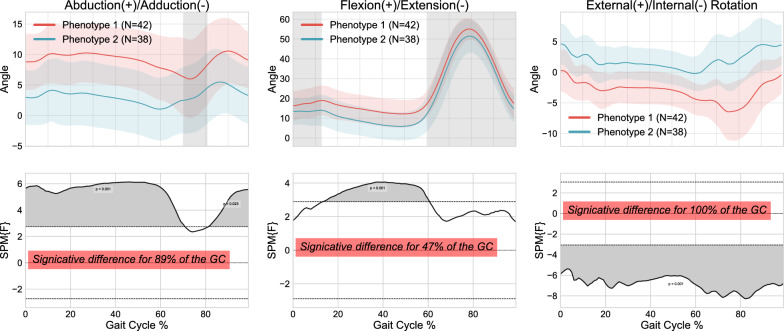
Kinematic phenotypes identified using K-means and standardization, with 1D-SPM statistical analysis using Principal Component Analysis- 2 clusters

**Fig. 8:**
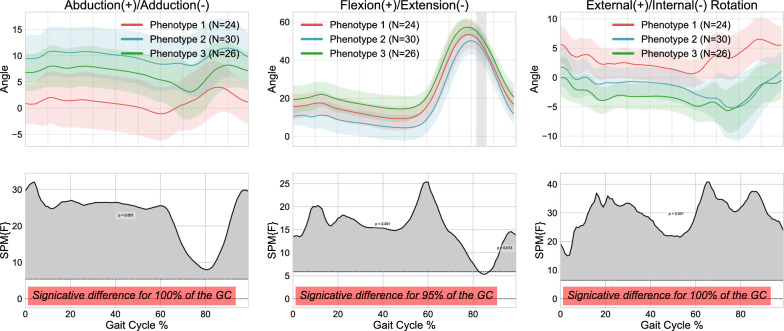
Kinematic phenotypes identified using K-means and standardization, with 1D-SPM statistical analysis using Principal Component Analysis- 3 clusters

The ICCs for the 2-cluster and 3-cluster approaches are detailed in Table 3, demonstrating a correlation between the various knee kinematic curves within the clusters, ranging from 0.36 to 0.874. The mean kinematic curves for the 2-cluster and 3-cluster approaches are displayed in Figs. 7 and 8 respectively. According to the SPM analysis, the two clusters largely differed throughout the entire gait cycle, with minimal differences occurring in the flexion/extension plane where they differed for 47% of the gait cycle. Considering the three clusters, the pairwise comparisons revealed more limited differences after Bonferroni correction. Indeed, for each pairwise comparison, the minimal differences corresponded to clusters differing for less than 40% of the gait cycle in at least one plane (i.e., 40% in flexion/extension between clusters 1 and 2, 12% in adduction/abduction between clusters 2 and 3, and 12% in flexion/extension between clusters 1 and 3).

Finally, differences in demographic, kinematic, and clinical data were assessed using the 2-cluster and 3-cluster approach, respectively. No significant differences were found between clusters in any approach for age, BMI, or sex (all $p-values$>0.05). The comparison of kinematic features revealed that, for the 2-cluster approach, 43 out of 69 features differed between clusters, mainly in the frontal (63.3% of the features from this plane) and transverse planes (81.8%; t test p-values $p \le 0.05$). For the 3-cluster approach, only 36 features differed between clusters. Regarding the clinical features, 3 out of the 6 features differed between clusters for the 2-cluster approach, while no features differed between the three identified clusters. Although similar trends were observed for the other clinical features, differences in the OKS-Overall, PCS-Rumination, and PCS-Helplessness scores were not significant (all $0.07< p < 0.29$,see Table 4).

**Table 3:** Intraclass Correlation Coefficients (ICC) for each cluster using Principal Component Analysis (PCA) with 2 clusters and PCA with 3 clusters

ICCs	PCA-2clusters		PCA-3clusters		
	Cluster1	Cluster2	Cluster1	Cluster2	Cluster3
Abduction/adduction	0.874	0.8	0.771	0.862	0.787
Flexion/extension	0.565	0.672	0.585	0.614	0.36
External/internal rotation	0.514	0.642	0.615	0.571	0.461

**Table 4:** Between-cluster differences on the 6 clinical features with the 2-cluster approach: Number of samples (N), mean value, standard deviation (SD), standard error (SE), p-value, and significance. The clinical features include Oxford Knee Score (OKS-overall), Pain Catastrophizing Scale (PCS overall), rumination, magnification, helplessness, and the Timed-Up-And-Go test (TUG-test)

	Group statistics						
Clinical features	Cluster	N	Mean	Std.deviation	Std. error mean	*p*-value	Significative Yes/No
OKS-Overall	1	39	38.23	7.28	1.17	0.29	No
	2	41	36.34	8.60	1.34		
PCS-Overall	1	32	20.85	13.39	2.37	0.05	Yes
	2	34	14.41	13.14	2.25		
PCS-Rumination	1	32	6.75	4.40	0.78	0.17	No
	2	34	5.206	4.58	0.79		
PCS-Magnification	1	32	5.000	3.41	0.60	0.01	Yes
	2	34	2.91	3.17	0.54		
PCS-Helplessness	1	32	9.10	6.51	1.15	0.07	No
	2	34	6.294	5.99	1.03		
TUG-test	1	38	10.83	3.71	0.60	0.01	Yes
	2	41	9.01	2.36	0.37		

## Discussion

The evaluation and comparison of ICCs, SPM, kinematic, and clinical differences between clusters suggested that the two-cluster approach was optimal, leading to the phenotypes displayed in Fig. 7. This approach captures a more distinct and representative characterization of the TKA candidates’ phenotypes, as the 3-cluster phenotype may introduce more complexity and potentially dilute the specific phenotypic patterns that are of interest in the context of TKA. Compared to Phenotype 2, Phenotype 1 (N=42 out of 80 patients) showed greater dynamic varus alignment ($9.9^{\circ }$ vs. $3.0^{\circ }$) and greater dynamic flexion contracture at heel strike ($16.3^{\circ }$ vs. $13.6^{\circ }$) and stance ($11.3^{\circ }$ vs. $5.7^{\circ }$). Interestingly, such kinematic features are known to be associated with poorer outcomes (i.e., satisfaction) post-TKA [27, 28]. Furthermore, these dysfunctions, or protective strategies to stabilize an unsteady knee in regard to stiff knee gait, appeared to be associated with pain catastrophizing, especially the magnification of pain, rather than its intensity or impact on the ability to perform ADLs. An accentuated varus alignment may also increase this lack of confidence in the knee and play a role in the poorer function reported on the TUG test. Thus, the determination of this particular phenotype may allow clinicians to identify patients who presented more severe dynamic malalignment, an accentuated protective strategy, and higher pain catastrophizing scores. Knowing that all these factors are associated with or predictors of poor outcomes post-TKA, identifying such patients could inform the need for personalized prehabilitation strategies to potentially improve surgical outcomes.

Regarding the methodological aspect of this study, the results provide insights into the use of data transformation methods and clustering algorithms for knee kinematics analysis. While the study did not directly compare its results with those of other state-of-the-art works, some comparisons can be drawn based on existing research. For instance, previous studies have used K-means clustering and rescaling methods, including standardization and min-max normalization, to analyze knee kinematics data [29, 30]. Similar to the current study, these studies found that the choice of data transformation can influence the clustering results. For instance, Visalkchi et al. [31] reported that no unique normalization procedure could yield better quality clusters for all datasets and that every dataset supported a specific method. DTW can be computationally expensive, especially for large datasets, and may not be the best choice when the data are linear or when the shapes of the data points are more consistent. Other studies have also used principal component analysis (PCA) for feature extraction and clustering of knee kinematics data [21, 22]. Similarly to the current study, authors reported that PCA can improve clustering performance by reducing the dimensionality of the data. In addition, authors in [32, 33] identified four distinct gait profiles in knee OA patients using machine-learning algorithms and principal component analysis. This highlights the potential for gait kinematics to reveal diverse movement patterns and inform clinical decisions. Their findings complement ours by demonstrating the clinical relevance of differentiating gait profiles based on kinematic representation.

However, the specific results may differ based on the dataset and clustering algorithm used. Overall, the findings of the current study align with previous research on knee kinematics analysis and provide further insights into the importance of normalization methods and feature extraction techniques for accurate clustering results. Through the analysis of gait patterns, it is possible to categorize TKA candidates into two distinct subgroups, each exhibiting unique kinematic characteristics, as well as clinical differences. This method of classification can provide a deeper understanding of the diverse biomechanical changes that occur in patients with KOA, helping clinicians to move toward more personalized care based on the patients’ affiliation.

## Conclusion

This study identified distinct kinematic phenotypes in TKA candidates using the K-means clustering technique. After assessing different transformation, representation, and data-point distance methods, the optimal clustering process consisted of a K-means algorithm using Euclidean distance, combined with standard deviation scaling and PCA. These phenotypes based on 3D kinematic data obtained with the $\hbox {KneeKG}^{\text {TM}}$ system allowed us to differentiate two profiles of patients who differed kinematically and clinically, reinforcing the relevance and potential of such an approach to provide surgeons with valuable insights for their choice of the most effective intervention. Findings may assist clinicians in more personalized treatment approaches which may ultimately improve surgical outcomes. While this approach may need to be applied on other databases to validate these phenotypes in TKA candidates, future work could also aim at exploring the potential of established machine learning techniques for the classification of the two clusters generated and to implement deep learning algorithms. Nonetheless, the results from this study highlight the feasibility of identifying kinematic phenotypes in KOA patients and provide a roadmap for future research in this domain.

## Data Availability

The participants data are confidential and cannot be shared in an open repository for unrestricted public access.

## Abbreviations

ADL: Activities of daily living
BMI: Body Mass Index
DTW: Dynamic time warping
ICC: Intraclass correlation coefficient
KneeKG: Knee Kinesiography exam
KOA: Knee osteoarthritis
OKS: Oxford Knee Score
PCA: Principal components analysis
PCS: Pain Catastrophyzing Scale
ROMs: Ranges of motion
SPM: Statistical Parametric Mapping
TKA: Total knee arhroplasty
TUG test: Timed-Up-And-Go test
